# Epigenetic Reprogramming of Muscle Progenitors: Inspiration for Clinical Therapies

**DOI:** 10.1155/2016/6093601

**Published:** 2015-12-29

**Authors:** Silvia Consalvi, Martina Sandoná, Valentina Saccone

**Affiliations:** ^1^IRCSS, Fondazione Santa Lucia, Via del Fosso di Fiorano 64, 00143 Roma, Italy; ^2^DAHFMO-Unit of Histology and Medical Embryology, University of Rome “La Sapienza”, Rome, Italy

## Abstract

In the context of regenerative medicine, based on the potential of stem cells to restore diseased tissues, epigenetics is becoming a pivotal area of interest. Therapeutic interventions that promote tissue and organ regeneration have as primary objective the selective control of gene expression in adult stem cells. This requires a deep understanding of the epigenetic mechanisms controlling transcriptional programs in tissue progenitors. This review attempts to elucidate the principle epigenetic regulations responsible of stem cells differentiation. In particular we focus on the current understanding of the epigenetic networks that regulate differentiation of muscle progenitors by the concerted action of chromatin-modifying enzymes and noncoding RNAs. The novel exciting role of exosome-bound microRNA in mediating epigenetic information transfer is also discussed. Finally we show an overview of the epigenetic strategies and therapies that aim to potentiate muscle regeneration and counteract the progression of Duchenne Muscular Dystrophy (DMD).

## 1. Introduction

Epigenetic regulation of chromatin structure is fundamental to achieve the activation or repression of transcriptional programs governing cell development and differentiation. Changing cell phenotype without affecting genotype, epigenetics controls the spatial and temporal regulation of gene expression that ensures the quality, stability, and heritability of cell identity. At least three systems, including DNA methylation, posttranslational histone tail modifications, and noncoding RNA, are currently involved in epigenetic regulation [1]. Epigenetic changes occur naturally in normal development and health but can also be influenced by several factors including aging and diseases. Indeed aberrant epigenetic control can cause abnormal activation or silencing of genes. Importantly epigenetic modifications are reversible and sensitive to the environment, having therefore the potential to be therapeutically manipulated. Thus, epigenetics is currently a hot topic for research and the number of studies relating to various models of epigenetic regulation is tremendously increasing. Moreover, advances in genome-wide technologies trying to elucidate epigenetic profiling (i.e., ChIP-seq, ChIA-PET, and Hi-C) hold the promise to deeply clarify the epigenetic control of cellular identity in health and disease.

Adult stem cells are candidate targets of epigenetic therapies toward repairing injured or diseased tissues, so they represent a key issue in regenerative medicine. In this context, skeletal muscle regeneration provides an insightful model for the study of the epigenetic events supporting the synchronized activation and repression of gene expression during stem cells differentiation. Indeed adult muscle stem cells remain in an embryonic-like state during development with the long-term ability to self-renewal and differentiate in response to injury [2]. A global genome reorganization allows activation, proliferation, and subsequent differentiation of quiescent progenitor muscle cells into functional multinucleated myofibers. Satellite cells are the main source of muscle stem cells (MuSCs) that regenerate adult skeletal muscles during postnatal life [3]. Intriguingly during aging or muscular disorders in which there is a chronic loss of skeletal muscle structure, the satellite cells function is compromised [4] even if their endogenous capacity to regenerate is not affected [5]. In fact it was demonstrated that the muscle environment is critical to permit effective muscle regeneration [6]. In particular the recently identified population of muscle interstitial cells, named fibroadipogenic progenitors (FAPs), plays a key role in supporting MuSCs activity and regeneration. However, in chronic muscle damage these cells lose their ability to support MuSCs mediated muscle regeneration and differentiate into fibroblasts and adipocytes [7–11]. An extensive analysis of the epigenome of these cells in healthy and diseased muscles is currently missing and would be crucial to better understand and pharmacologically manipulate changes that affect their regeneration activity.

The most severe neuromuscular disease is the Duchenne Muscular Dystrophy (DMD), a rare X-linked genetic disease caused by mutations in the dystrophin gene. DMD is characterized by a rapid progression of muscle degeneration that leads to the loss of ambulation and death within the second decade of life. In DMD the unbalanced regeneration of muscles exposed to continuous waves of degeneration leads to replacement of contractile myofibers with fibrotic and fatty tissue [12, 13]. Nowadays there is not available cure for dystrophic patients and treatment is restricted to strategies that counteract the progression of the disease. The only therapy is limited to using corticosteroids as drugs to improve muscle strength. A huge number of studies for the treatment of the muscular disease are arising and some of them are undergoing clinical investigation. Gene and cell-therapies, acting to repair the genetic defect, represent the most promising curative approach in the treatment of DMD but are still far from clinical translation [14].

Otherwise, pharmacological approaches that target the pathological consequences of the genetic defect are easy prompt to clinical practice translation. Actually, the pharmacological therapy for DMD includes nitric oxide (NO) administration, insulin-like growth factor 1 (IGF-1) stimulation, and myostatin inhibition in way to increase skeletal muscle mass; otherwise, therapies leading the inhibition of the transforming growth factor-beta (TGF*β*) pathway, modulation of nuclear factor-*κ*B (NF-*κ*B), and tumor necrosis factor-*α* (TNF-*α*) signalling are used to reduce fibrosis and inflammation in muscle [15].

However, the major limitation of manipulating target pathways consists in the lack of selectivity resulting in undesired side effects. Thus, regenerative medicine is providing novel strategies developing several epigenetic drugs aimed to manipulate the chromatin targets of individual signalling pathways. In the context of DMD, Histone Deacetylase Inhibitors (HDACi) are emerging as promising treatment to increase the functional and morphological recovery of dystrophic muscles [16–18]. Most of the beneficial effects of HDACi arise from their ability to activate a microRNA-SWI/SNF based epigenetic network in FAPs that redirects their lineage commitment from a fibroadipogenic toward a myogenic fate [19].

MicroRNAs (miRs) belong to the small noncoding RNAs family and are known to control numerous biological processes representing the most prevalent regulatory mechanism of mRNA availability in cells [20]. Apart from their role in regulating cell-autologous epigenetic events, miRs are involved also in cell-to-cell communication being involved in epigenetic regulation of recipient cells. miR shuttle between cells appears to be preserved and mediated by extracellular vesicles (i.e., exosomes) that are emerging as potent genetic transfer agents [21]. Interestingly stem cell-derived extracellular vesicles appear to be naturally equipped to mediate tissue regeneration and recent evidence suggests their therapeutic potential for targeted delivery of exogenous miRs [22].

In this review, we will focus on the principal epigenetic regulatory mechanisms underpinning skeletal muscle regeneration and their potential manipulation to develop pharmacological therapies for the treatment of DMD.

## 2. Chromatin-Modifying Enzymes: Epigenetic Writers and Erasers Regulating Cell Epigenome

The temporally regulated gene expression that controls pluripotency and differentiation is achieved by highly coordinated epigenetic events that ensure lineage commitment and cell fate determination. Epigenetic regulation of chromatin structure is fundamental to the activation or repression of specific transcriptional programs and is mainly controlled by chromatin-modifying enzymes that induce DNA methylation, posttranslational histone tail modifications, and nucleosome remodelling.

DNA methylation is a heritable, yet reversible, epigenetic modification that plays a central role in transcriptional repression. DNA methyltransferases (DNMTs) catalyze the transfer of a methyl group from cofactor* S*-adenosylmethionine to carbon 5 of the cytosines (5mC) that typically reside within a CpG dinucleotide. Regions of high CpG density, known as CpG islands, are typically devoid of DNA methylation [23]. Conversely, genes regulated by methylation usually contain low CpG density promoters and are demethylated and expressed in a cell-type-specific manner during differentiation [24–26]. This process is well illustrated during skeletal muscle cell fate commitment and differentiation. During development, pluripotent cells show a progressive loss of methylation leading to muscle stem cells with a unique DNA methylation signature associated with its specialized functions. Specific-myogenic factors such as MyoD and Myogenin are activated in a demethylation-dependent manner driving the activation of the myogenic program (reviewed in [27]). Simultaneously, myogenesis is accompanied by DNA methylation of pluripotency and developmental genes (i.e., Hox genes) [28]. Seminal works demonstrated that treatment with 5-azacytidine, a potent inhibitor of DNA methylation, triggers myogenic differentiation in nonmuscle cells, linking for the first time* MyoD *tissue-specific demethylation and cell fate commitment [29–31]. DNA demethylation may also provide a transcriptionally poised state of muscle fiber genes that would be activated during differentiation, upon the acquisition of transcription factors and positive histone marks.

Indeed changes in DNA methylation and histone modifications strongly cooperate to achieve the global genome reorganization of progenitor cells necessary to establish myogenic identity, proliferation, and subsequent differentiation.

Satellite cells represent the main source of stem cells for adult muscle regeneration. Following muscle injury, they are readily activated and induced to proliferate and differentiate in multinucleated myofibers [32, 33]. The myogenic lineage in satellite cells is determined by the expression of Pax3 and Pax7 genes, while the expression of basic helix-loop-helix Myogenic Regulatory Factors (MRFs; MyoD, Myf5, Myogenin, and MRF4) in cooperation with myocyte enhancer factor-2 (MEF2) family proteins confers their ability to form differentiated myofibers [34]. Satellite cells activation is reflected in drastic changes at specific chromatin regions via the action of chromatin-modifying enzymes [35].

There are several classes of posttranslational histone modifications (i.e., phosphorylation, acetylation, methylation, and ubiquitylation) that affect chromatin structure and accessibility [36].

Histone acetylation has generally been linked to transcriptional active chromatin and is dynamically regulated by the opposing activities of histone acetyltransferases (HATs) and histone deacetylases (HDACs). A large amount of work illustrated the fundamental role of HATs and HDACs in regulating muscle development and differentiation. HATs catalyse the transfer of acetyl groups to lysine residues of histones, resulting in the relaxation of chromosomal DNA permissive for transcription. The histone acetyltransferases p300/CBP and PCAF activate muscle gene expression by acetylation of MyoD and modulation of its recruitment at target loci [37]. Interestingly recent studies have highlighted the ability of MyoD to preset the chromatin landscape of myoblasts for the activation of muscle-specific genes. Indeed genome-wide binding of MyoD has been associated with HATs recruitment and regional histone acetylation [38], while MyoD-bound distal enhancers have been linked to the recruitment of additional transcription factors and the regional enrichment of H3K4 monomethylation (H3K4me1) and H3K27 acetylation (H3K27ac), two typical markers of active enhancers [39].

HDACs function to reverse histone acetylation, causing chromosomal DNA condensing and preventing the unscheduled transcriptional activation of muscle-specific genes in undifferentiated cells. There are currently 18 known human HDACs grouped into four classes [40]. Classes I, II, and IV HDACs are zinc-dependent proteins, while class III HDACs require NAD^+^ [41].

Interestingly, class I HDACs (HDAC1 and HDAC2) show constitutive nuclear localization and preferentially associate with MyoD [42], while class II HDAC members (HDAC4 and 5) shuttle between the nucleus and the cytoplasm and are dedicated repressors of MEF2-dependent transcription [43, 44]. Upon differentiation, displacement of HDACs from the chromatin of target genes correlates with the hyperacetylation at muscle loci and activation of muscle gene transcription (i.e., Myogenin and Myosin heavy chain) [45].

Given the importance of the balance between acetylation and deacetylation in regulating muscle gene transcription, HDACi are emerging as promising drugs to manipulate the regenerative potential of stem cells in diseased muscle [15] (see below). Despite the general assumption that HDAC inhibition would indiscriminately cause a global hyperacetylation in all organs and tissues, several studies revealed a surprising selective effect of HDACi on embryonic and adult stem cells [46] and in particular on genes with bivalence or with preexisting activator marks [15, 47]. Accordingly genome-wide Chip-seq analysis in myoblasts showed that the large majority of HDACi induced genes were involved in the myogenic differentiation program (i.e., Myosin 7 (MyH7), Enolase 3 (ENO3), and Myomesin 1 (MYOM1)) and showed bivalent (42%) or active (57%) epigenetic marks [48]. This data indicates that, in myoblasts, HDACi enforce and anticipate the expression of genes that are normally induced during differentiation. A bivalent chromatin structure builds an epigenetic signature that identifies genes poised for transcription typically enriched in stem cells. Poised genes show bivalent promoters marked by the presence of both active and repressive histone methylation marks [49, 50].

Methylation is linked to both active and inactive chromatin regions depending on the specific histone and lysine residue that is targeted. In particular, tri-methylation of lysine 4 on histone H3 (H3K4me3) is associated with transcriptionally active promoters, while tri-methylation of lysine 27 (H3K27me3) leads to chromatin condensation [51].

Differentiated cells usually resolve bivalent promoters into an active or repressive state [52, 53] and become resistent to HDACi treatments. Indeed HDACi have been shown to potentiate myogenesis and the gene expression profile selectively in proliferating myoblasts and not in terminally differentiated myotubes. As differentiation precedes, muscle specific genes (i.e., Myogenin and MCK) gradually lose H3K27me3 and gain H3K4me3 [54]. Conversely, progenitor-specific transcription factors (i.e., Pax7) require H3K27me3-mediated epigenetic repression for myotube maturation [55]. This process is finely regulated by the activity of two classes of histone lysine methyltransferases: the Polycomb group proteins responsible of H3K27me3 for epigenetic silencing and the Trithorax group (TrxG) which activates gene transcription catalyzing H3K4me3.

Dynamic changes in the epigenetic landscape of muscle progenitors during differentiation are coordinated by extracellular signals that specifically target the activity and recruitment of chromatin modifier enzymes. For instance, the regeneration-activated p38 signalling targets multiple components of the myogenic transcriptosome that is assembled on the chromatin of muscle genes in response to locally released regeneration cues. p38*α*/*β* kinases phosphorylate MEF2D mediating the recruitment of the Trithorax enzymatic subunit Ash2L to the chromatin of muscle genes [56]. Concomitantly p38*α* kinase promotes the phosphorylation of EZH2, the enzymatic subunit of the Polycomb Repressive Complex 2 (PRC2), targeting Pax7 promoter for repression [55]. Finally p38 signalling promotes the recruitment of the chromatin remodelling SWI/SNF complex to the regulatory regions of MyoD-target muscle genes by the phosphorylation of BAF60c [57].

SWI/SNF complex comprises two mutually exclusive enzymatic sub-units (the ATPases Brg1 and Brm) and several Brg1/Brm associated factors (BAFs) [58]. In particular, three alternative variants of Baf60 sub-unit (BAF60a, BAF60b, and BAF60c) confer the affinity for tissue-specific transcription factors regulating lineage determination in many cell types [19, 59–61]. BAF60c is essential to activate both skeletal and cardiac muscle programs [57, 61], while BAF60a and BAF60b activate alternative lineages, including lipid metabolism [62]. During embryo myogenesis, the negative regulation of BAF60a and BAF60b leads in progenitor cells the activation of a BAF60c-mediated muscle differentiation program [63].

**Figure 1:**
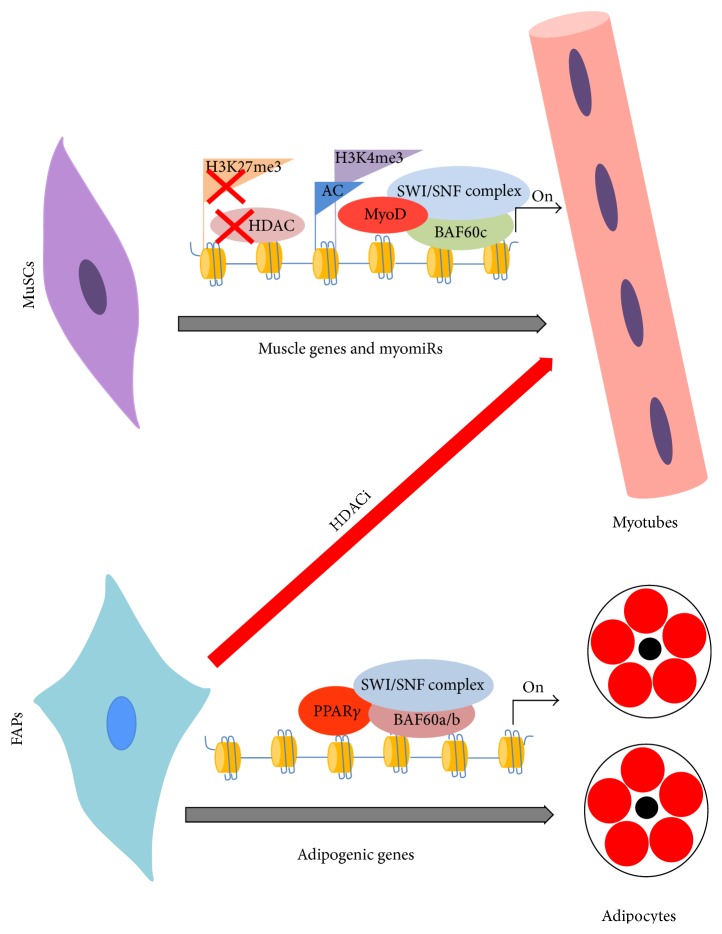
Epigenetic reprogramming of MuSCs and FAPs during differentiation. MuSCs adopt a chromatin permissive structure on muscle genes in which MyoD and BAF60c-based SWI/SNF complex promote transcription (on the top). FAPs differentiation into adipocytes is mediated by BAF60a/b-based SWI/SNF complex (on the bottom). HDACi treatment in dystrophic muscles activates a myomiR/MyoD/BAF60c network that, switching the BAF60 subunits assembled in the SWI/SNF complex, reprograms FAPs toward the acquisition of a myogenic phenotype.

Intriguingly our recent study demonstrated that BAF60 selection can drive lineage determination in a population of fibro-adipogenic progenitors (FAPs) resident in skeletal muscles. Favouring BAF60c incorporation in SWI/SNF complex at expense of BAF60a/b directs the switch from the fibro-adipogenic to the myogenic lineage reducing fibrosis and fat deposition in dystrophic muscles (Figure 1) [19, 64]. These data suggest that therapeutic approaches aim to selectively target the combinatorial assembly of the SWI/SNF complex could be used to manipulate cell fate determination in several disorders.

## 3. Non-Coding RNAs as Epigenetic Regulators of Gene Expression

A novel emerging level of gene expression regulation is mediated by non-coding RNAs (ncRNAs): functional RNA molecules not translated into proteins, composite of structural and regulatory RNAs. ncRNAs are divided by their size into* long non-coding RNA* (lncRNAs) greater than 200 nucleotides to over 100 kb in length and* small non-coding RNA* (sncRNAs) with a non-coding transcript long less than 200 nucleotides [65].

LncRNAs localize both in the nucleus and cytoplasm and have roles in chromatin remodelling, transcription, intracellular trafficking and post-translational processes controlling cell identity and lineage commitment [66, 67]. LncRNAs located in the nucleus regulate transcription recruiting chromatin-modifying enzymes or interacting with RNA sequences to influence their splicing. Many nuclear lncRNAs associate with EzH2/PRC2 and control the formation of nuclear compartments (i.e., speckles, para-speckles, polycomb bodies) [68, 69]. lncRNAs identified in* cytoplasm* regulate protein localization, mRNA translation and stability. Intriguingly it was recently described their role as sponge for miRNAs: reducing miRNAs levels, they inhibit the miRNA-mRNA mediated target degradation [66]. lncRNAs strongly regulate MuSCs differentiation. The muscle-specific linc-MD1 manages the time of muscle differentiation acting as a sponge to sequester miR-133 and miR-135 that regulate the expression of MAML1 and MEF2C, pro-myogenic transcription factors [66]. lncRNAs transcribed from MyoD enhancer (Enhancer RNAs (eRNAs)) regulate also MyoD and Myogenin expression [70].

sncRNAs family include transfer RNAs (tRNAs) and ribosomal RNAs (rRNAs), as well as microRNAs (miRNAs), Piwi-interacting RNAs (piRNAs), small interfering RNAs (siRNAs), small nuclear RNAs (snRNAs) [65]. Recent studies suggest the existence of thousands of ncRNAs, many of them involved in the epigenetic regulation of development, physiology, tissue regeneration and disease [71, 72]. The most studied small non-coding RNAs are the miRNAs, molecules containing about 22 nucleotides, expressed in eukaryotes and found well conserved in plants and animals. miRNAs regulate numerous biological processes inhibiting translation of their target mRNA and also mediating their degradation through recognition of imperfect complementary sites, usually located in the 3′-untranslated regions [73, 74]. It seems that miRNAs regulate the expression of more than 50% of mammalian genes making them the most prevalent regulatory mechanism of mRNA availability [20, 75, 76].

miRNAs may fine tuning distinct processes targeting specific epigenetic regulators: DNA methylases, PRC components, Histone Deacetylases and chromatin remodelling complexes members [77]. Given their consistent epigenetic role, miRNAs are important regulators of embryonic and adult myogenesis [78] controlling MuSCs quiescence, proliferation and differentiation [79, 80]. For instance, Rando's group identified in a microarray expression study, about twenty quiescence-specific miRNAs that actively maintain the quiescent state of satellite cells (i.e., miR-489 that targets the oncogene Dek) and 351 miRNAs regulating satellite cells activation [81].

One crucial step for MuSCs activation is miR-31 downregulation that allows Myf5 translation in myoblasts [82]. Myf5, together with MyoD, leads the activation of miR-133a/b that inhibit the adipogenic regulator PRDM16 preventing muscle progenitors cells commitment to adipose cell fate [83, 84]. miR-133 controls also myoblasts proliferation acting as SRF regulator [85] During myogenesis, miR-1, miR-29 and miR-206 target HDAC4 promoting the activity of the myogenic transcriptional elements Mef-2 and MRFs [86]. MRFs in turn regulate the expression of miR-1, miR-133a/b and miR-206, muscle specific miRNA defined as “myomiRs”. Finally miR-1 and miR-206 control Pax3/7 repression [87] while miR-26a targets the Ezh2 methyltransferase, to allow muscle differentiation [54, 88].

Different studies demonstrated that miRNAs could modulate the composition of SWI/SNF chromatin remodelling complexes in a way to epigenetically reprogram cell fate determination. Crabtree showed a microRNA mediated switching of chromatin-remodelling complexes in neural development: miR-9 and miR-124 target BAF53a sub-unit driving differentiation of progenitor cells into neurons [89]. Similarly our group identified in muscle interstitial FAPs an analogous miR-based mechanism that regulates the balance toward myogenic versus alternative fates (fibro-adipogenesis). In FAPs myomiRs (miR-1,2as, miR-133a and miR-206) favour the composition of the pro-myogenic BAF60c-SWI/SNF complex by targeting the alternative BAF60a and BAF60b variants [19]. Similarly in Embryonic muscle progenitors myomiRs negatively regulate BAF60a/b to promote the BAF60c-SWI/SNF complex [63].

Interestingly miRNAs derived from various tissues and organs, being stable and resistant to nuclease digestion, are easily detectable in both plasma and serum and may serve as diseases biomarker. Indeed circulating miRNAs profile dynamically change in many diseases such as cancer, myocardial infarction, heart failure, myotonic Distrophy type I and DMD [90–95]. MyomiRs for instance, have been identified in serum of muscular dystrophy animal models and patients where they are passively released as a consequence of myofibers degeneration and breakdown. Their putative active role is still unkown and currently they are proposed as novel diagnostic markers of disease progression. Indeed myomiRs detection in serum is inversely correlated to muscle health, representing a more sensible biomarker than the commonly used Creatine Kinase (CK) [94, 96].

## 4. Extracellular Vesicles for Genetic Information Transfer and Cell Phenotype Modulation

Extracellular vesicles are emerging as potent sources of genetic information transfer between cells and are involved in regulating stem cell plasticity via epigenetic reprogramming and their ability to alter gene regulatory networks [21]. Cell-derived vesicles such as exosomes and microvesicles possess the capability to mediate intercellular communication by fusing with the plasma membrane of recipient cells and subsequently delivering their cargo, consisting of functional proteins, mRNAs and miRNAs able to modulate gene expression and cell phenotype [97]. Exosomes are homogenous small particles, usually 30 to 100 nm in size, of endosomal origin. Microvesicles, instead, constitute a larger and heterogeneous population of extracellular vesicles, 50 to 1000 nm in size, and are directly produced through the plasma membrane budding [22].

Multiple cell types have been described to release vesicles in extracellular medium, including mesenchymal cells, adipocytes, fibroblasts, immune cells and myoblasts. Little is known about vescicles regulation of MuSCs in health and diseased muscles. However several studies reporting muscle-exosomes are emerging [98–100]. Myoblasts and myotubes use exosome clustered miRNAs as “endocrine signals” to control important signaling pathways (i.e., Wnt signaling pathway) for muscle homeostasis and regeneration. MiRNAs secreted in exosome by myotubes are functionally able to silence the HDAC Sirt1 in myoblasts, controlling their commitment to differentiation [88]. Muscle behaviour is also influenced by vesicles released from different sources, like mesenchymal stem cells. Indeed it has been recently shown that miRNAs (i.e., miR-494 and myomiRs) released in exosomes from mesenchymal stem cells promote muscle regeneration following injury by enhancing myogenesis and angiogenesis [101]. Indeed exosomes appear to be naturally equipped to mediate tissue regeneration and their cargo constitute a rapid response, protected by the oxidative environment, to initiate tissue repair [102]. Vesicles from mesenchymal stem cells were found to confer therapeutic benefit in a range of different diseases: kidney [103–105] and hepatic injuries [106] myocardial ischaemia and infarction [107–109] and peripheral arterial disease [110]; this “regenerative” effect is mainly due to the ability of extracellular-vesicles to induce phenotypic changes in local stem cells through epigenetic reprogramming to stimulate tissue repair and regeneration [111]. Notably, the transfer of tissue-specific mRNAs, miRNAs and protein-based transcription factors through the extracellular microvesicles was shown to induce phenotype change in bone marrow cells when co-cultured with cells derived from various tissues (brain, heart, liver and lung) [112–114].

Extracellular vesicles mediate communication even between distally located cells and tissue and can be found in many biological fluids including blood, saliva, urine, and breast milk [22]. For instance tumor cells can induce apoptosis in distal skeletal muscles via exosome assembled miR-21, which signals through the Toll-like 7 receptor (TLR7) on myoblasts to promote cell death and cancer cachexia [115].

Given their ability to be readily isolated from most body fluids, circulating miRNAs packed into exosomes are emerging as useful biomarkers to determine the development and progression of various diseases. Moreover, their natural role in transferring genetic material both locally and systemically has inspired pharmacological strategies to exploit these vesicles as therapeutic agents via the introduction of exogenous genetic cargoes such as siRNA (see below).

## 5. Epigenetic Therapies and Future Perspectives for Muscle Regeneration in DMD

In last years, regenerative medicine focused on the study of plasticity of stem cells epigenome and the recent findings lead the researchers to concentrate on strategies aimed to reprogram the stem cell fate in numerous diseases.

Myogenesis is coordinated by a complex interplay between epigenetic events that are crucial to control lineage determination and differentiation of adult stem cells. Basic research and recent studies of next generation sequencing are clarifying the fine epigenetic regulation of myogenesis and which are the epigenetic players that create changes in the epigenome opening new therapeutic options in muscle diseases as DMD.

HDACi are considered as the first generation of epigenetic drugs with proven clinical efficacy in the treatment of some lymphoid malignancies [116] and are now in clinical trials for a number of other diseases including DMD. Indeed preclinical studies in dystrophic mice (mdx) showed their ability to alleviate both morphological and functional consequences of the primary genetic defect [16, 17]. The current availability of HDACi in clinical practice gave the opportunity for an immediate translation of these drugs into pharmacological treatments of DMD in human patients. The HDACi ITF2357 (Givinostat) represents the first epigenetic drug included into a study therapy for DMD.

Givinostat has already been tested in pediatric populations and received an Orphan Drug Designation by EMA for the treatment of systemic-onset juvenile idiopathic arthritis (SOJIA) [117, 118]. This knowledge encouraged its traslation into a phase I/II clinical trial with children affected by DMD (ClinicalTrials.gov identifier: NCT01761292). After one year of treatment Givinostat efficacy has been monitored showing very promising results on muscle histology and functionality without severe adverse effects on children health; thus the trial has been prolonged for a second year. Obviously this study requires defining the activity of Givinostat in long-term treatments to assess its persistent effect in dystrophic muscles and to monitor possible adverse events.

The functional characterization of the recently identified epigenetic network that determines the ability of HDACi to promote regeneration of dystrophic muscles, at expense of fibrosis and fat deposition, highlights the role of FAPs as key cellular mediators of HDACi activity and DMD progression [18, 19].

FAPs are multipotent mesenchymal cells located in muscle interstitium with the ability to proliferate and support satellite cells mediated muscle regeneration in response to local injury or disease. However, beyond their beneficial role, FAPs have been shown to be the major source of fibro-adipocytes in degenerating muscles [9, 10].

Importantly we have demonstrated that treatment with HDACi at early stages of DMD induces in FAPs a myogenic fate at expense of their fibro-adipogenic lineage. HDACi de-repress a latent myogenic program by activating a MyoD/BAF60c/myomiR network that leads muscle differentiation. Indeed HDACi induce MyoD and BAF60c expression, two core components of the myogenic transcriptional machinery, and up-regulate myomiRs (miR-1.2, -133 and -206), which target the alternative BAF60 variants A and B. Switching of the BAF60 sub-units assembled in the SWI/SNF complex reprograms FAPs toward the acquisition of a pro-myogenic phenotype. However the progressive impairment of the integrity of this network prevents HDACi efficacy at late stages of DMD. Indeed, with the progress of the disease FAPs becomes resistant to HDACi and acquire a constitutive fibro-adipogenic lineage replacing the muscle loss with fatty and fibrotic tissues [18]. Importantly, transplantation of “young” FAPs into muscles of  “old” dystrophic mice, restored the ability of HDACi to promote regeneration at advanced stages of disease [18]. This suggests that a powerful future therapeutic strategy will be to epigenetically reprogram aged FAPs with selective delivery of Baf60c and myomRs. In this context the natural ability of exosomes to transfer material both locally and systemically encourage the possibility of exploiting these vesicles for therapeutic purposes.

While these data provide new insight into the molecular pathogenesis of DMD and therapeutic approaches to delay the disease, they also highlight the potential of miRs detection as clinical biomarkers of disease progression. The increase of circulating myomiRs in the peripheral blood of dystrophic patients correlates with the severity of the disease, suggesting that myomiR quantification in blood of DMD patients might represent a sensible diagnostic and prognostic marker [64, 94]. On the other hand our recent data, show a great increase of FAPs derived myomiRs in muscle interstitium of mdx mice after HDACi exposure suggesting an inverse correlation between local and circulating myomiRs [19]. This suggests that detection of muscular (local) versus circulating myomiRs could provide a novel more accurate biomarker for diagnosis of DMD progression and efficacy of therapeutic drugs [64].

**Figure 2:**
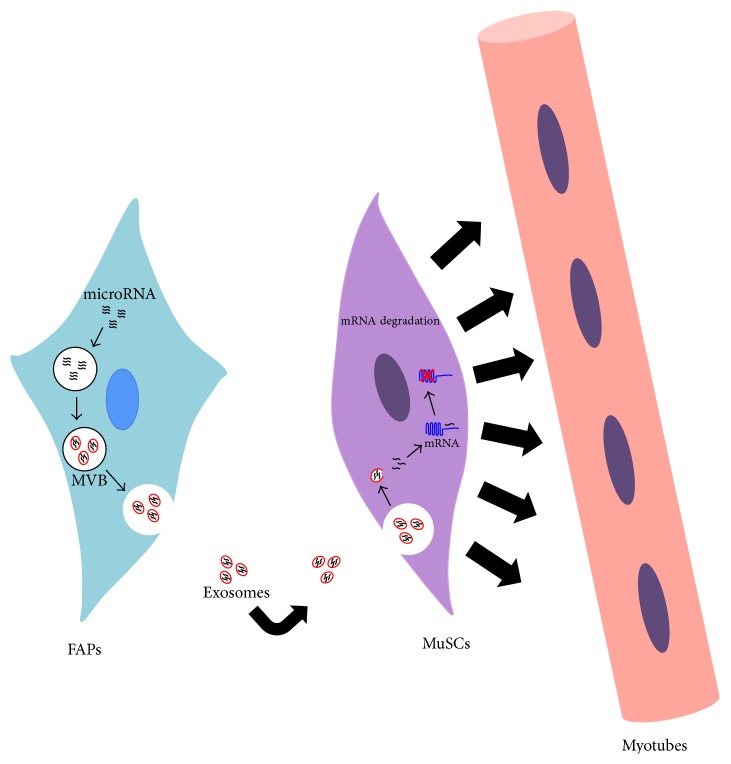
Exosomes as putative mediators of the functional interaction between FAPs and MuSCs. In this model, exosomes released by activated FAPs support myoblasts differentiation through a mechanism by which their cargo of miRNA can be transferred to MuSCs.

MiR stability in extracellular environment seems to be preserved by vesicles budding and intriguingly myomiRs have been detected* in vitro* in exosomes released by mesenchymal cells to support myoblasts differentiation [101]. An analogous mechanism is probably involved* in vivo* between FAPs and MuSCs to promote muscle regeneration (Figure 2). It would be important to investigate if this functional cross-talk mediated by exosome is somehow affected in muscle disorders. Furthermore these data strongly encourage the possibility to re-engineer naturally derived exosomes for DMD epigenetic therapy.

## 6. Conclusions

In the last years, great advances have been made in the comprehension of the epigenetic mechanisms regulating, via chromatin organization, different transcriptional programs. The functional characterization of the variety of epigenetic regulations in healthy and disease states has the prospect to identify novel targets for epigenetic-based therapies.

HDACi represent the first generation of epigenetic drugs. Their clinical efficacy is currently being tested in a phase I/II clinical trial on children affected by DMD. The pro-regenerative effects of HDACi are mediated by FAPs, a population of muscle-resident stem cells. However, dystrophic muscles at late stages of the disease are resistant to HDACi-induced beneficial effects. This unresponsiveness might be due to a decreased chromatin plasticity of FAPs caused by epigenetic silencing pathways. The identification of the epigenetic players preventing HDACi responsiveness at advanced stages of DMD will be crucial to devise new personalized and selective strategies to re-establish HDACi sensitivity. In this context, a comprehensive epigenetic mapping of the chromatin landscape of key populations involved in muscle regeneration is becoming urgent to identify in the near future both therapeutic effectiveness and inclusion criteria of DMD patients to epigenetic therapy.

Exosome-bound miRNAs are emerging as a crucial mechanism to transfer epigenetic information between cells. New evidence showing the therapeutic relevance of these vesicles in both unmodified and modified forms make them attractive therapeutic agents for further study. Moreover detection of specific miRNAs secreted in muscle interstitium and blood of dystrophic patients holds the promise to develop new painless methodologies, less invasive than classic biopsy, such as blood sampling or fine needle aspiration techniques, to diagnose DMD.
